# A novel assay for the introduction of the vinyl ether double bond into plasmalogens using pyrene-labeled substrates

**DOI:** 10.1194/jlr.D080283

**Published:** 2018-03-14

**Authors:** Ernst R. Werner, Markus A. Keller, Sabrina Sailer, Daniele Seppi, Georg Golderer, Gabriele Werner-Felmayer, Raphael A. Zoeller, Katrin Watschinger

**Affiliations:** Division of Biological Chemistry, Biocenter* Medical University of Innsbruck, Innsbruck, Austria;; Division of Human Genetics,† Medical University of Innsbruck, Innsbruck, Austria; and; Department of Physiology and Biophysics,§ Boston University School of Medicine, Boston MA

**Keywords:** ether lipid, plasmanylethanolamine desaturase, phosphatidyl ethanolamine, lyso-phosphatidyl ethanolamine

## Abstract

Plasmanylethanolamine desaturase (PEDS) (EC 1.14.99.19) introduces the 1-prime double bond into plasmalogens, one of the most abundant phospholipids in the human body. This labile membrane enzyme has not been purified and its coding sequence is unknown. Previous assays for this enzyme used radiolabeled substrates followed by multistep processing. We describe here a straight-forward method for the quantification of PEDS in enzyme incubation mixtures using pyrene-labeled substrates and reversed-phase HPLC with fluorescence detection. After stopping the reaction with hydrochloric acid in acetonitrile, the mixture was directly injected into the HPLC system without the need of lipid extraction. The substrate, 1-*O*-pyrenedecyl-2-acyl-*sn*-glycero-3-phosphoethanolamine, and the lyso-substrate, 1-*O*-pyrenedecyl-*sn*-glycero-3-phosphoethanolamine, were prepared from RAW-12 cells deficient in PEDS activity and were compared for their performance in the assay. Plasmalogen levels in mouse tissues and in cultured cells did not correlate with PEDS levels, indicating that the desaturase might not be the rate limiting step for plasmalogen biosynthesis. Among selected mouse organs, the highest activities were found in kidney and in spleen. Incubation of intact cultivated mammalian cells with 1-*O*-pyrenedecyl-*sn*-glycerol, extraction of lipids, and treatment with hydrochloric or acetic acid in acetonitrile allowed sensitive monitoring of PEDS activity in intact cells.

Plasmalogens make up about 18% of all phospholipids in the human body and are especially abundant in membranes of the brain and of certain immune cells (1). They have been suspected to be involved in the development of Alzheimer’s disease, Parkinson’s disease, and atherosclerosis (2). Plasmalogens are glycerophospholipids that contain a 1-alkenyl residue. The crucial 1-prime ether bond, which gives this lipid class special chemical, biophysical, and biochemical characteristics, is introduced by plasmanylethanolamine desaturase (PEDS) (EC 1.14.99.19). This labile membrane enzyme has not been purified and a gene encoding this enzyme has not been assigned yet, currently making it one of 94 human orphan enzymes (3).

Previous assays of PEDS have used radiolabeled substrates, lipid extraction, and two-dimensional thin-layer chromatography with scraping off bands and scintillation counting to assay the enzyme (4–6). When the radiolabel was placed in position 2 of the glycerol by chemical synthesis, a solvent partitioning system could be employed following lipid extraction and a two-step chemical cleavage procedure (7).

We present here a much more straight-forward method for the quantification of PEDS activity using substrates with fluorescent 1-*O*-pyrenedecyl side chains and reversed-phase HPLC with fluorescence detection. Once stopped, the reaction mixture can be directly injected to the reversed-phase HPLC system without the need for a lipid extraction procedure or scraping off bands. With this method, we achieve, for the first time, a quantitative comparison of PEDS activities in several mouse tissues and in commonly used human and mammalian cell lines. Presumably due to the laborious procedures required so far, no data on these activities can be found in the literature. The HPLC system we employ here is also useful for the analysis of pyrene-labeled plasmalogens following incubation of intact cells with 1-*O*-pyrenedecyl-*sn*-glycerol, as well as for the isolation of 1-*O*-pyrenedecyl-labeled glycerophospholipids from cells.

**Figure 1** introduces the (bio)chemical reactions and the formulae of the compounds mentioned in this work. As will be detailed below, the lyso-substrate, 1-*O*-pyrenedecyl-*sn*-glycero-3-phosphoethanolamine [I], is acylated to the actual substrate, 1-*O*-pyrenedecyl-2-acyl-*sn*-glycero-3-phosphoethanolamine [II], in the incubation mixture. PEDS then introduces the crucial vinyl ether double bond to yield 1-*O*-(1′Z)-pyrenedecenyl-2-acyl-*sn*-glycero-3-phosphoethanolamines [III]. These can be cleaved by hydrochloric acid, but not by acetic acid, to pyrenedecanal [IV].

**Fig. 1.**
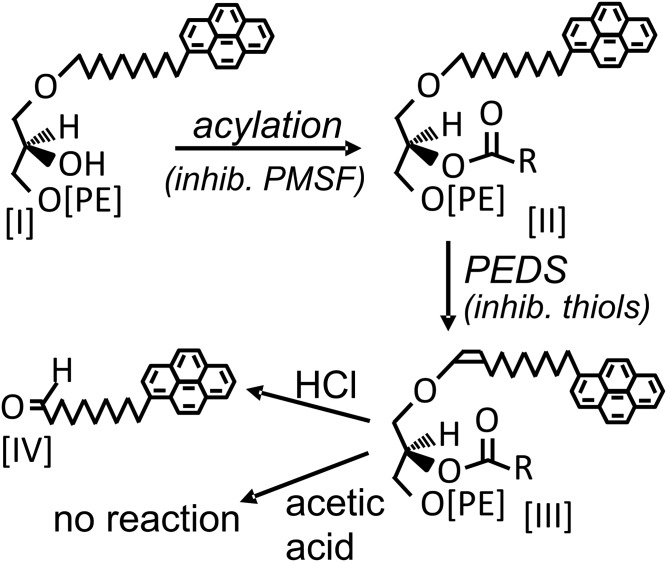
Reaction scheme of the PEDS assay carried out with lyso-substrate. The lyso-substrate, 1-*O*-pyrenedecyl-*sn*-glycero-3-phosphoethanolamine [I], is first acylated, presumably by CoA-independent transacylase, a reaction we find to be inhibited by PMSF and SKF 98625. The resulting 1-*O*-pyrenedecyl-2-acyl-*sn*-glycero-3-phosphoethanolamine [II] is heterogenous in that different residues (R) are attached. At least three different major peaks were separated by HPLC (see Fig. 3A). The PEDS reaction then introduces the vinyl ether double bond to yield 1-*O*-(1′Z)-pyrenedecenyl-2-acyl-*sn*-glycero-3-phosphoethanoalmines [III], i.e., pyrene-labeled plasmalogens. The amount of pyrene-labeled plasmalogens formed is then quantified by measuring pyrenedecanal released from the reaction products by HCl. To control for any nonplasmalogen-derived pyrenedecanal, the same enzyme incubation mixture is treated with acetic acid and analyzed in parallel. PE, phosphoethanolamine; R, fatty acid side chains.

## MATERIALS AND METHODS

### Reagents and cell lines

The 1-*O*-pyrenedecyl-*sn*-glycerol was obtained from Otava Ltd. (Vaughan, Ontario, Canada), pyrenedecanal from Ramidus (Lund, Sweden), and diethyl 7-(3,4,5-triphenyl-2-oxo-2,3-dihydro-imidazol-1-yl)heptane-phosphonate (SKF 98625) from KareBay Biochem, Inc. (Monmouth Junction, NJ). All other chemicals were from Sigma (Vienna, Austria), Roth (Karlsruhe, Germany), or Serva (Heidelberg, Germany). The cell lines, RAW-108 and RAW-12, had been prepared in previous work in one of our laboratories (R.A.Z.) (8). All other cell lines were from ATCC (Manassas, VA).

### Cell culture and mouse tissues

Cell lines were grown in media recommended by the supplier containing 10% fetal bovine serum (except for MOLT4, which required 20% fetal bovine serum) at 37°C in a humidified 5% (v/v) CO_2_ atmosphere. The employed media were very low endotoxin DMEM (FG1445, Biochrom, Berlin, Germany) for A431, HEK 293T, A549, CaCo-2, HEP G2, RAW264.7, RAW-12, and RAW-108; DMEM (Gibco 31966, Thermo-Fisher, Waltham, MA) for SK-N-SH and SK-HEP1; DMEM (Gibco 21885) for SH-SY5Y; RPMI (R8758, Sigma) for HL-60, K562, THP-1, MOLT-4, U937, DLD-1, and Jurkat E6-1; EMEM30-2003 (ATCC) for MCF-7; MEM-Alpha (Gibco 22561) for T24; and F12K (Gibco 21127) for CHO-K1. Additional supplements used were insulin (10 μg/ml; Sigma I9278) for MCF-7; 50 μM mercaptoethanol for THP-1; 2.5 g/l glucose, 10 mM HEPES, and 1 mM sodium pyruvate for HL-60 and Jurkat. Cells were harvested, washed with PBS, and dry pellets immediately snap-frozen in liquid nitrogen and stored at −80°C until analyzed. C57BL/6N mice were obtained from the animal facility of the Medical University of Innsbruck, which is approved by the National Committee for Animal Care of the Austrian Federal Ministry of Science, Research, and Economy. For PEDS tissue distribution, three female and three male 10-week-old C57BL/6N mice were euthanized by cervical dislocation. Seven-day-old mice were anesthetized with isoflurane before decapitation. Tissues were excised, immediately snap-frozen in liquid nitrogen, and stored at −80°C until analysis.

HPLC analysis was done on an Agilent 1200 HPLC system equipped with a thermostatted autosampler, UV-Vis and fluorescence detectors, a column thermostat, and a Merck L1200 fraction collector. At a flow rate of 1 ml/min and an injection volume of 10 μl, a Zorbax Eclipse XDB-C8 4.6 × 50 mm, 3.5 μm particle size column (Agilent Technologies, Vienna, Austria) was eluted with 10 mM potassium phosphate buffer (pH 6.0) containing 79% (v/v) methanol for 3 min, followed by a linear gradient to 100% methanol at 10 min. Methanol (100%) was held until 15 min, and the column was then equilibrated to starting elution buffer until 17 min. Pyrene-labeled compounds were detected by UV absorption at 340 nm and by fluorescence (excitation 340 nm, emission 405 nm). Plasmalogen-derived pyrenedecanal liberated upon HCl treatment was monitored by comparison to an external synthetic standard. The same HPLC system was also used for the purification of the substrate, 1-*O*-pyrenedecyl-2-acyl-*sn*-glycero-3-phosphoethanolamine, and the lyso-substrate, 1-*O*-pyrenedecyl-*sn*-glycero-3-phosphoethanolamine, using a 50 or 100 μl injection volume and water instead of 10 mM potassium phosphate buffer as component of the eluents.

### Preparation of the substrate 1-*O*-pyrenedecyl-2-acyl-*sn*-glycero-3-phosphoethanolamine [II]

Twelve 75 cm^2^ flasks containing RAW-12 cells (8), which have a deficiency in PEDS and in peroxisomal ether lipid precursor biosynthesis, were incubated with 10 μM 1-*O*-pyrenedecyl-*sn*-glycerol for 48 h and the cells were collected in 30 pellets in 2 ml tubes. For each aqueous 100 μl pellet, lipids were extracted with two times 500 μl chloroform/methanol 2:1 (v/v) (9); the combined organic phases were evaporated to dryness and each extract was taken up in 100 μl methanol-acetonitrile 1:1 (v/v), incubated for 30 min at 37°C with the addition of 100 μl acetonitrile/2 M HCl (895:105, v/v) to eliminate residual vinyl ether bonds, and finally injected to the HPLC system in preparative mode (100 μl injection volume). The fluorescent fractions eluting from 11.3 to 12.2 min were collected, the solvent was evaporated with a Centrivac vacuum concentrator (Heraeus, Hanau, Germany), and the residual solid was dissolved in methanol (material equivalent to 4 cell pellets in 170 μl). The concentration of the labeled substrate [II] was calculated by relating the area of the peaks at UV 340 nm and the area of a peak of a known concentration of synthetic pyrenedecanal.

### Preparation of the lyso-substrate 1-*O*-pyrenedecyl-2-lyso-*sn*-glycero-3-phosphoethanolamine [I]

To 170 μl of 1-*O*-pyrenedecyl-2-acyl-*sn*-glycero-3-phosphoethanolamine substrate [II], prepared as described above, 42 μl of chloroform and 20 μl of NaOH were added and the mixture was incubated for 1 h at 40°C to cleave off the 2-acyl residue. Then, 80 μl of aqueous 500 mM KH_2_PO_4_ and 291 μl of chloroform were added, mixed, centrifuged, the organic phase collected, and the aqueous phase reextracted with 500 μl of chloroform-methanol (2:1 v/v). The organic phases were combined, the solvent was removed, and the residue was dissolved in 100 μl acetonitrile/ethanol 1:1 (v/v). The resulting solution was then subjected to purification with the HPLC system (injection volume 50 μl) and the single fluorescent peak of the lyso-substrate [I] was collected and pooled, concentrated, and taken up in methanol. The concentration was determined by comparison of peak areas at UV 340 nm to synthetic pyrenedecanal. In a typical preparation, the initial cellular extract contained 294 nmol pyrene-labeled phospholipids (100%); after the first HPLC purification, the yield for the substrate [II] was 193 nmol (66%), finally resulting in 105 nmol (36%) of the lyso-substrate [I] after NaOH treatment and the second HPLC purification.

PEDS assay was done with microsomal fractions of cells or tissues. For preparation of microsomes, cell pellets or tissue pieces were homogenized in 400–800 μl of 0.1 M Tris HCl and 0.25 M sucrose (pH 7.6) by use of an Ultra Turrax (IKA, Staufen, Germany) and centrifuged for 10 min at 4°C and 3,000 *g*. For tissue samples, protease inhibitors tested not to impair PEDS activity (aprotinin, 1 μg/ml; pepstatin A, 1 μM; trypsin inhibitor, 100 μg/ml; iodacetamide, 2 mM; and leupeptin, 1 μg/ml) were added to the homogenization buffer. The supernatant was then centrifuged for 30 min at 20,000 *g* and 4°C, the pellet resuspended in 40–100 μl Tris HCl-sucrose buffer, and protein concentration determined with a Bradford assay (BSA standard; Bio-Rad, Vienna, Austria). The solution was then diluted with 3.5 mg/ml BSA to an equivalent of 0.5 mg microsomal protein per milliliter. The assay mixture contained, in a total volume of 25 μl (final concentrations): 0.1 M Tris HCl (pH 7.2); 0.1 mg/ml catalase (2,000–5,000 U/mg; Sigma C1345); 1 mM NADPH; 2 mM EDTA; 2 μM pyrene-labeled lyso-substrate [I] or substrate [II]; and 0.15 mg/ml microsomal protein. The reaction was started by addition of the diluted microsomal preparation. Following incubation for 30 min at 37°C, the reaction mixture was divided into two 10 μl portions and the reaction was stopped by addition of 30 μl of acetonitrile/2 M HCl (895:105 v/v) to one 10 μl aliquot to cleave the vinyl ether bond, or by addition of 30 μl of acetonitrile/2 M acetic acid (895:105 v/v) to the other 10 μl aliquot as a control, respectively. The mixtures were incubated for 30 min at 37°C to complete the cleavage reaction. All samples were centrifuged at 20,000 *g* for 5 min; 10 μl were injected to the HPLC system and the amount of pyrenedecanal formed from plasmalogen cleavage quantified. When reagents barely soluble in water were tested for their influence on PEDS activity, they were dissolved in DMSO and the control incubations adjusted to the same final DMSO concentrations, which never exceeded 1% (v/v). To determine the recovery of activity from added microsomes, 0.075 mg/ml of test microsomes and 0.075 mg/ml of spiking microsomes (RAW-108 for tissues and A431 for cells) were incubated in the assay mixture and the activity compared with the result of 0.075 mg/ml of spiking microsomes alone.

### Quantification of formation of pyrene-labeled plasmalogens in intact cells

**Fig. 2.**
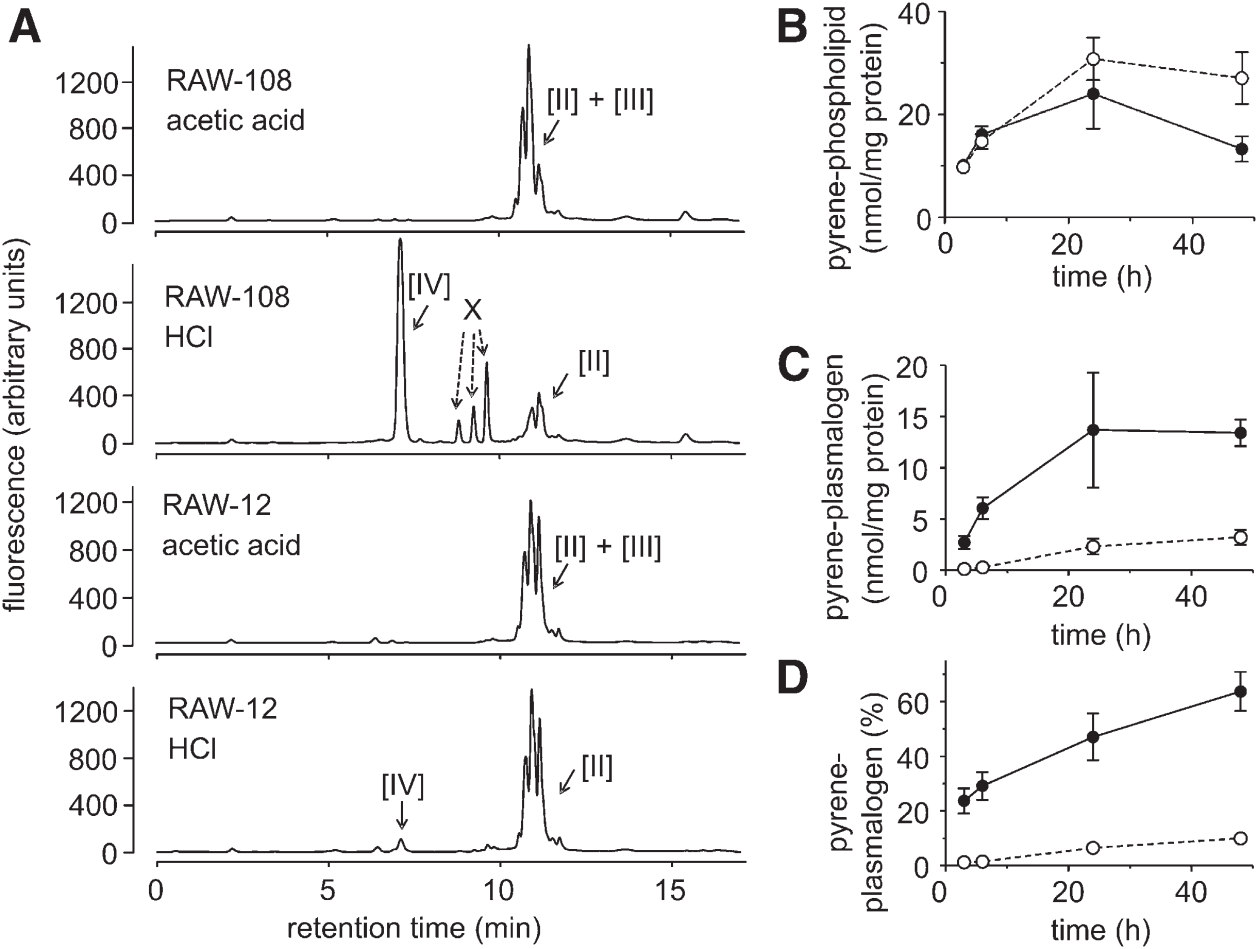
Formation of pyrene-labeled plasmalogens from 1-*O*-pyrenedecyl-*sn*-glycerol by intact cells. A: Chromatograms of lipid extracts of RAW clones 12 and 108. Cells were cultivated for 24 h in the presence of 5 μM 1-*O*-pyrenedecyl-*sn*-glycerol, treated with HCl (to cleave the vinyl ether bond) or with acetic acid (which leaves the vinyl ether bond intact). RAW-12 has a defect in PEDS activity, which is intact in RAW-108 (8) (see text for details). The peak labeling corresponds to Fig. 1 ([II], 1-*O*-pyrenedecyl-2-acyl-*sn-*glycerophospholipids; [III], 1-*O*-(1′Z)-pyrenedecenyl-2-acyl-*sn*-glycerophospholipids; [IV], pyrenedecanal). X denotes peaks that originate from the reaction of pyrenedecanal with unknown compounds present in the lipid extract (see text for details). B: Time dependence of formation of pyrene-labeled phospholipids in RAW264.7 cells (filled circles, solid line) and RAW-12 cells (open circles, dashed line). Cells were cultivated for the indicated times in the presence of 5 μM 1-*O*-pyrenedecyl-*sn*-glycerol, harvested, lipids extracted, and the extracts treated with HCl (which cleaves the vinyl ether bond) or acetic acid (which leaves the vinyl ether bond intact), respectively. The amount of pyrene-labeled phospholipids was calculated from the area of retention times between 10 and 12 min in extracts treated with acetic acid. The mean ± SEM for four independent experiments is shown. C: Time dependence of formation of pyrene-labeled plasmalogen in RAW264.7 cells (filled circles, solid line) and RAW-12 cells (open circles, dashed line). The amount of pyrene-labeled plasmalogen was calculated from the amount of pyrenedecanal plus its derivative peaks (X) liberated upon HCl treatment and related to cellular protein. Free pyrenedecanal was not present, as judged by analysis of extracts treated with acetic acid only. The mean ± SEM for four independent experiments is shown. D: Percentage of pyrene-labeled 2-acyl-glycerophospholipids with vinyl ether double bond in RAW264.7 (filled circles, solid line) and RAW-12 (open circles, dashed line). Chromatograms of the extracts prepared for B and C were evaluated for the amount of aldehyde [including its derivatives (X)] formed in relation to the sum of aldehyde formed plus pyrene-labeled glycerophospholipids stable to HCl (retention times 10–12 min). The mean ± SEM for four independent experiments is shown.

Cells were seeded in 6-well plates and the culture medium supplemented with 5 or 10 μM of 1-*O*-pyrenedecyl-*sn*-glycerol. Cells from a single well of a 6-well plate were sufficient to perform the analysis. After 3–48 h, cells were collected and an aliquot was kept for the determination of cellular protein. Lipids were extracted with chloroform/methanol 1:1. All organic phases were collected and dried. The dried lipid extracts were taken up in 100 μl acetonitrile/ethanol 1:1 (v/v). Aliquots of the extract (12.5 μl) were mixed with 50 μl of acetonitrile/2 M HCl (895:105 v/v) or with 50 μl of acetonitrile/2 M acetic acid (895:105 v/v), respectively. After 15 min on ice, the incubation mixtures were centrifuged for 5 min at 20,000 *g* and 4°C, and 10 or 20 μl aliquots were injected to the HPLC system as described above. The extent of formation of pyrene-labeled plasmalogens was quantified by the formation of pyrenedecanal upon HCl treatment, or by the difference between HCl- and acetic acid-treated extracts of the area of glycerophospholipids eluting at 10–12 min. Peaks originating from the reaction of pyrenedecanal with components of the lipid extract in the acidic medium (“X” in Fig. 2A) were included into the aldehyde quantification, assuming that the fluorescence of these derivatives was equal to the free aldehyde.

### Determination of plasmalogen content of cells and tissues

Tissue pieces were homogenized in 400–1,000 μl of PBS with an Ultra-Turrax (IKA, Staufen, Germany); protein concentration was determined with a Bradford assay (BSA standard; Bio-Rad) and was adjusted to a concentration of 2.5 mg/ml. Cell pellets were homogenized in 200 μl of PBS by shaking them with glass beads, yielding homogenates of 0.8–4 mg/ml protein. Two hundred microliters of these aqueous homogenates were extracted twice with 500 μl of chloroform/methanol (2:1 v/v); the organic phases were combined and evaporated to dryness. Lipid extracts were dissolved in 100 μl of acetonitrile/ethanol (1:1 v/v) by shaking for 10 min at 37°C. Ten microliters of lipid extracts were then derivatized by mixing them either with 40 μl of dansylhydrazine [Sigma 03334, 0.45 mg/ml in acetonitrile/2 M aqueous HCl (930:70 v/v)], which cleaves plasmalogens to the respective aldehydes and yields the corresponding dansylhydrazides, or with 40 μl of dansylhydrazine [0.45 mg/ml in acetonitrile/2 M aqueous acetic acid (930:70 v/v)], which leaves the plasmalogens intact and derivatizes free aldehydes only. The method was calibrated by the external synthetic standards, 1-(1Z-octadecenyl)-2-oleoyl-*sn*-glycero-3-phosphoethanolamine (Avanti Polar Lipids, Alabaster, AL) and hexadecanal (10). The resulting mixtures were directly injected to the HPLC system described above and the dansylhydrazine derivatives eluting from 8 to 10 min were quantified by the fluorescence peak area (excitation 340 nm, emission 525 nm). Plasmalogen levels were corrected for free aldehydes, which were typically below 1% of the corresponding plasmalogen levels.

## RESULTS

### Incorporation of 1-*O*-pyrenedecyl side chains into plasmalogens in intact cells

As a first step, we checked to determine whether PEDS would accept 1-*O*-pyrenedecyl-glycerophosphoethanolamines as substrates. For this purpose, we fed 1-*O*-pyrenedecyl-*sn*-glycerol to RAW264.7 cell clones, which in previous work had been selected for a deficiency in peroxisomal plasmalogen precursor synthesis (RAW-12 and RAW-108). Clone RAW-12 has an additional deficiency in PEDS activity (8). We then treated lipid extracts of these cells with acetonitrile/HCl (to cleave the vinyl ether bond yielding the respective aldehydes) or with acetonitrile/acetic acid as a control, which leaves the vinyl ether bond intact. Chromatograms are shown in **Fig. 2A**. Both cell clones formed a comparable amount of pyrene-labeled phospholipids [II + III], which eluted in the range of 10–12 min, as indicated by the chromatograms of the acetic acid-treated controls (top chromatogram and third chromatogram). Upon treatment with HCl, however, only in extracts of RAW-108 (which have an intact PEDS activity), major amounts of pyrenedecanal [IV] were released and 69.0 ± 0.81% (mean ± SEM, n = 3) of the pyrene-labeled phospholipids were degraded (second chromatogram). In RAW-12, in contrast, only 3.25 ± 0.052% (mean ± SEM, n = 3) of 1-*O*-pyrenedecyl-containing phospholipids were cleaved to the aldehyde (fourth chromatogram). We chose acetonitrile rather than methanol as solvent for the acid treatment to avoid the formation the respective dimethyl acetal, which did not separate as well as the aldehyde from the more complex phospholipids. Peaks labeled with X in the RAW-108 HCl chromatogram are adducts formed by pyrenedecanal released from plasmalogens with unidentified components contained in the lipid extract. We confirmed this by incubating pyrenedecanal in the acidic stop solution with extracts of cells not treated with pyrene-containing compounds (chromatograms not shown). These peaks are therefore included when quantifying the amount of aldehyde formed.

Figure 2B shows a time course for the accumulation of pyrene-labeled phospholipids in parent RAW264.7 and the RAW-12 clone that had been incubated with the pyrene-labeled ether lipid precursor, 1-*O*-pyrenedecyl-*sn*-glycerol. At the end of the incubation, RAW-12 accumulated even more pyrene-labeled phospholipids than RAW264.7. Due to its deficiency in PEDS, however, RAW-12 accumulated significantly fewer fluorescent plasmalogens than RAW264.7 (Fig. 2C), resulting in a much lower percentage of plasmalogen formed (Fig. 2D).

### A PEDS assay for microsomal fractions of cells and tissues

We then isolated the 1-*O*-pyrenedecyl-labeled phospholipids accumulating in RAW-12 upon feeding with 1-*O*-pyrenedecyl-*sn*-glycerol to prepare a substrate for an in vitro enzyme incubation assay. Lipids extracted by chloroform/methanol were incubated with hydrochloric acid to remove any residual vinyl ether bonds and the 1-*O*-pyrenedecyl-glycerophospholipid [II]-containing fractions were collected. Because it had been described that the lyso-substrate [I] is superior to the substrate [II] in performance in the assay (6), we then hydrolyzed the 2-acyl groups by NaOH to obtain the pyrene-labeled lyso-substrate [I].

**Fig. 3.**
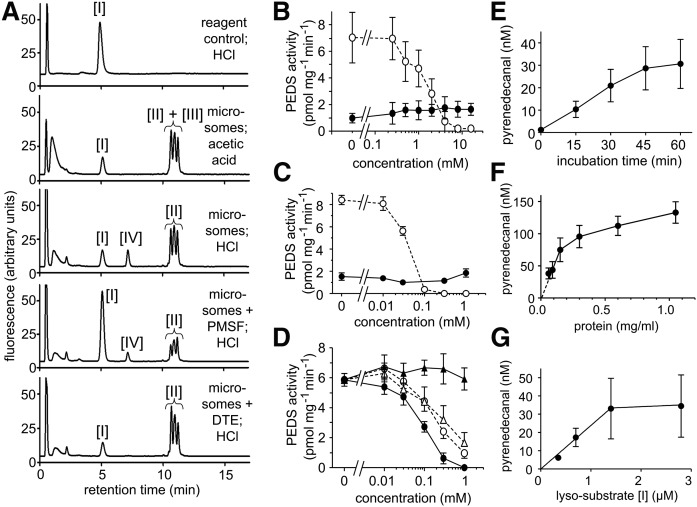
PEDS assay with microsomal fractions. A: Chromatograms of typical enzyme incubations using microsomes from RAW-108 as the source for the enzymatic activity. The labeling of the peaks corresponds to Fig. 1 ([I], lyso substrate 1-*O*-pyrenedecyl-*sn*-glycero-3-phosphoethanolamine; [II], 1-*O*-pyrenedecyl-2-acyl-*sn-*glycerophospholipids; [III], 1-*O*-(1′Z)-pyrenedecenyl-2-acyl-*sn*-glycerophospholipids; [IV], pyrenedecanal) (see text for details). B: Inhibition of the enzymatic activity by PMSF. Assay incubations with substrate [II] (filled circles, solid line); assay incubations with lyso-substrate [I] (open circles, dashed line). The mean ± SEM for three independent experiments is shown. C: Inhibition of the enzymatic activity by SKF 96825. Assay incubations with substrate [II] (filled circles, solid line); assay incubations with lyso-substrate [I] (open circles, dashed line). The mean ± SEM for three independent experiments is shown. D: Inhibition of the enzymatic activity with thiol reagents. The enzymatic assay was performed with the lyso-substrate [I]. DTT (filled circles, solid line); DTE (open circles, dashed line); mercaptoethanol (open triangles, dashed line); TCEP (filled triangles, solid line). The mean ± SEM for three independent experiments is shown. E: Dependence of pyrenedecanal formed on the incubation time using the lyso-substrate [I] (mean ± SEM for three independent experiments). F: Dependence of the amount of pyrenedecanal formed on the amount of microsomal protein using the lyso-substrate [I] (mean ± S.E.M for three independent experiments). G: Dependence of the amount of pyrenedecanal formed on the concentration of lyso-substrate [I] employed (mean ± SEM for three independent experiments).

**Figure 3A** shows typical chromatograms of the enzyme incubation assay with RAW-108 microsomal fractions using the lyso-substrate [I]. As is seen from the reagent control in the first chromatogram of Fig. 3A, the pyrene-labeled lyso-substrate [I] eluted as a single peak at about 5 min retention time, and no aldehyde was released from the reagent by HCl. Microsomes heated for 5 min at 80°C, incubated for 30 min at 37°C with the assay mixture, and stopped with HCl or with acetic acid gave similar chromatograms (not shown). When an enzyme incubation mixture with microsomes containing the activity was stopped with acetic acid (second chromatogram), it was apparent that much of the lyso-substrate [I] had been acylated to pyrene-labeled glycerophospholipids eluting at 10–12 min, which contained compounds [II] and [III]. When HCl instead of acetic acid was used to stop the incubation (third chromatogram), pyrenedecanal [IV] was released, corresponding to the amount of vinyl ether double bond formed, thus allowing quantification of PEDS activity. The fourth and the fifth panel show chromatograms of incubations with an additional two compounds commonly added to enzyme incubation mixtures, which we found to inhibit the reaction. In the fourth panel, an incubation containing 2 mM PMSF is shown. This protease inhibitor is also known to inhibit acyl-CoA independent transacylase (11). Both the formation of pyrenedecyl-2-acyl-glycerophospholipids [II] by acylation and the introduction of the double bond to yield plasmalogen [III], and hence the amount of the aldehyde released, were inhibited. In the fifth chromatogram, an incubation with 1 mM dithioerythritol (DTE) stopped with HCl, the desaturation and, as a consequence, the aldehyde formation was also greatly impaired, but the acylation reaction from the lyso-substrate [I] to the substrate [II] was not altered (compare also Fig. 1). Control incubations with addition of the thiol at the end of the incubation period confirmed that the desaturation reaction itself, not the release of the aldehyde from the plasmalogen by the HCl treatment, was impaired by the thiol (not shown).

When the substrate [II] was used instead of the lyso-substrate [I], approximately 7-fold lower desaturase activity was observed (Fig. 3B). PMSF did not alter the activity of the substrate [II] even at very high concentrations, whereas it inhibited the reaction of the lyso-substrate [I] with an IC_50_ of 1.6 ± 0.25 mM (mean ± SEM, n = 3; Fig. 3B). SKF 98625, a more specific inhibitor of the acyl-CoA independent transacylase [EC 2.3.1.147 (12)], was much more effective in inhibiting acylation and, hence, desaturation (IC_50_ = 0.040 ± 0.001 mM, mean ± SEM, n = 3) when using the lyso-substrate [I], again with no effect on substrate [II]-induced desaturation (Fig. 3C). We then compared inhibition of desaturase activity by the thiol compounds, DTT, DTE, and mercaptoethanol, and the disulfide bond reducing reagent, tris(2-carboxyethyl)phosphine (TCEP; Fig. 3D), using the lyso-substrate [I]. While the reduction of disulfide bonds by TCEP did not have an effect, all three tested thiols strongly inhibited the desaturase reaction at 1 mM concentrations, with a clear trend toward strongest action of DTT, followed by comparable action of DTE and mercaptoethanol (Fig. 3D).

Formation of plasmalogen-derived aldehyde increased almost linearly up to 45 min (Fig. 3E). Therefore, we chose 30 min as the standard incubation time. When using microsomal protein final concentrations of more than 0.15 mg/ml, a plateau was reached and plasmalogen formation did not further increase even in presence of a several-fold excess of microsomal protein (Fig. 3F). Therefore, we used 0.15 mg/ml microsomal protein in our assays. Lyso-substrate [I] concentrations above 1.4 μM could not further increase the activity (Fig. 3G). Therefore, we chose 2 μM as the standard assay concentration. NADPH was required for the reaction. To test for potential additional soluble factors required for the activity, we supplemented the microsomal preparations with concentrated soluble supernatant, with a variety of redox active cofactors (tetrahydrobiopterin, FAD, FMN, glutathione, ascorbic acid), or with dyes transferring reducing equivalents from NADPH to the active site of other enzymes (phenazine methosulfate, 1-methoxy-5-methylphenazinium methyl sulfate, 2,6-dichlorphenol-indophenol, methylene blue) or various metal ions (Fe^2+^, Fe^3+^, Mn^2+^, Zn^2+^, Cu^2+^). With all of these compounds, only inhibitory effects, if any, were observed. Also, addition of commonly used detergents (Triton X-100, n-dodecyl β-D-maltoside, sodium cholate, Tween 20, Fos-choline 12, 1,2-diheptanoyl-*sn*-glycero-3-phosphocholine) did not stimulate, but rather inhibited, the activity (data not shown).

### PEDS activities of microsomes of mouse tissues and of mammalian cell lines

**Fig. 4.**
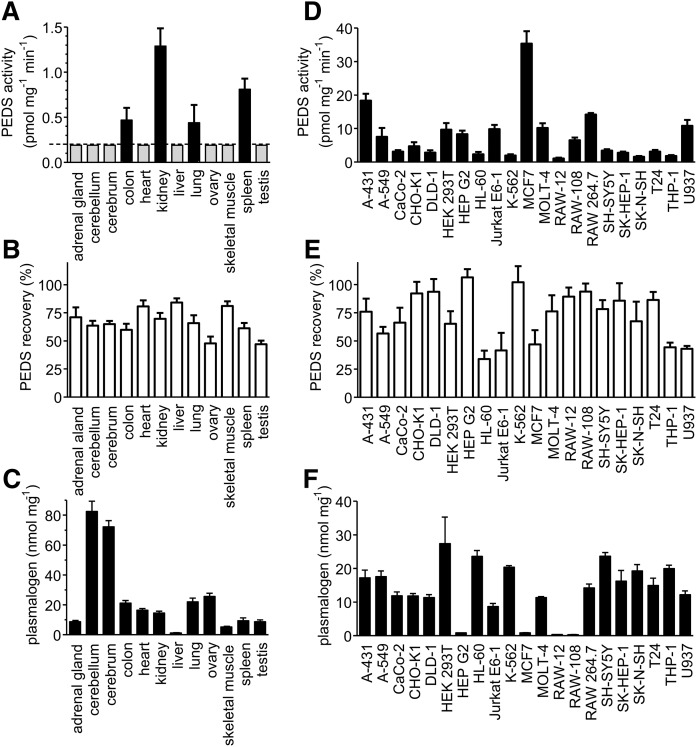
PEDS activities and plasmalogen levels in mouse tissues and cultured mammalian cells. A: PEDS levels in mouse tissues. Tissues from three male and three female 10-week-old C57BL/6N mice were homogenized in the presence of protease inhibitors. Microsomes were prepared and subjected to the PEDS assay using the lyso-substrate [I]. Because there was no gender difference, the mean ± SEM of six determinations is shown, with the exception of three for ovaries and testis, respectively. The dashed line indicates the detection limit of the method and gray bars indicate activities below the detection limit. B: Recovery of spiking of tissue microsomes with RAW-108 microsomes. Tissue microsomes (0.075 mg/ml) and RAW-108 microsomes (0.075 mg/ml) were incubated with the lyso-substrate [I] and compared with the PEDS activity with 0.075 mg/ml RAW-108 alone. The mean ± SEM for five to six independent measurements is shown, with the exception of three for ovary and testis, respectively. C: Plasmalogen levels in tissue homogenates. Plasmalogen levels were determined by HPLC quantification of dansyl-hydrazide derivatives as is detailed in the Materials and Methods section. The mean ± SEM for three independent measurements is shown D: PEDS activity in cell lines. Cell lines were cultivated under standard conditions, harvested, and microsomes prepared and subjected to the enzymatic assay using the lyso-substrate [I]. The mean ± SEM for three to eleven independent measurements is shown. E: Recovery of spiking of tissue microsomes with RAW-108 microsomes. A431 was used for spiking cell extracts instead of RAW-108 due to the one order of magnitude higher activity levels observed in cells as compared with mouse tissues. Microsomes (0.075 mg/ml) of the indicated cells and 0.075 mg/ml A431 microsomes were used with the lyso-substrate [I] and the recovery calculated. The mean ± SEM for two to eleven independent measurements is shown. F: Plasmalogen levels in cell homogenates. Plasmalogen levels were determined by HPLC quantification of dansyl-hydrazide derivatives as is detailed in the Materials and Methods section. The mean ± SEM for three independent measurements is shown.

We then used our assay to examine PEDS activities in tissues of 10-week-old C57BL/6N mice (**Fig. 4A**). The activities we found were rather low, with the highest activities in the kidneys (1.29 ± 0.20 pmol mg^−1^ min^−1^) followed by spleen (0.81 ± 0.12 pmol mg^−1^ min^−1^), colon (0.47 ± 0.13 pmol mg^−1^ min^−1^), and lung (0.44 ± 0.20 pmol mg^−1^ min^−1^; all values mean ± SEM, n = 6). Activities in all other tissues tested were below the limit of detection (gray bars in Fig. 4A). To check for potential inhibiting substances in the microsomal preparations, the reactions were spiked with microsomes from RAW-108 cells and the recovery was calculated. For this purpose, 0.075 mg/ml of test microsomes and 0.075 mg/ml of RAW-108 microsomes were incubated together in the assay mixture, and the PEDS activity compared with that of 0.075 mg/ml of RAW-108 microsomes alone. Recovery was found within a range of 50–75% for all tissues (Fig. 4B), thus ruling out that the differences in the tissue activities came from different levels of endogenous inhibiting substances. The kidney was also the only organ in which the activity could be clearly detected using the substrate [II] rather than the lyso-substrate [I] (not shown). Because a strong age dependence had been reported for PEDS in rat brain (13), we measured the activity in the cerebellum and cerebrum of 7-day-old mice (two female, one male) and found activities of 0.70 ± 0.15 pmol mg^−1^ min^−1^ for cerebellum and 1.38 ± 0.11 pmol mg^−1^ min^−1^ for cerebrum (mean ± SEM, n = 3). We also measured plasmalogen levels in all examined tissues (Fig. 4C). These were highest in the cerebrum and cerebellum and intermediate in all other organs, with the lowest levels in the liver. Remarkably, the levels did not correlate with PEDS activities.

Activities in microsomes from cultured cells were more than one order of magnitude higher than for mouse tissues (Fig. 4D). To observe potentially inhibiting endogenous substances, we did spiking and recovery experiments in a manner similar to the experiments with mouse tissues described above. Due to the higher activity observed in cells, however, we used microsomes from A431 cells rather than from RAW-108 cells for spiking, because A431 displayed higher activities. Recovery ranged from about 50% to 100% (Fig. 4E). Thus, like in the mouse tissues, the differences in the PEDS levels appeared not to be primarily dependent on endogenous inhibitory substances. As with the mouse tissues, plasmalogen levels of various cell lines (Fig. 4F) did not correlate with PEDS activities (Fig. 4D). RAW-12 and RAW-108, for example, showed equally reduced plasmalogen levels (0.35 ± 0.01 nmol mg^−1^ and 0.32 ± 0.02 nmol mg^−1^, respectively, mean ± SEM, n = 3 each) as compared with RAW264.7 (14.2 ± 2.00 nmol mg^−1^, mean ± SEM, n = 3), despite clearly different PEDS activities (1.09 ± 0.24 pmol mg^−1^ min^−1^ for RAW-12, 6.55 ± 0.78 pmol mg^−1^ min^−1^ for RAW 108, mean ± SEM, n = 4 for RAW-12, n = 10 for RAW-108). MCF7 cells with the highest PEDS activity (35.3. ± 3.75 pmol mg^−1^ min^−1^, mean ± SEM, n = 5) had only a tiny plasmalogen content (0.86 ± 0.12 nmol mg^−1^, mean ± SEM, n = 3). Hep G2 with an intermediate PEDS activity (8.36 ± 1.00 pmol mg^−1^ min^−1^, mean ± SEM, n = 4) was also almost devoid of plasmalogens (0.84 ± 0.10 nmol mg^−1^, mean ± SEM, n = 3).

## DISCUSSION

PEDS introduces the crucial vinyl ether bond into plasmalogens, one of the major phospholipid classes in our body. The introduction of this bond makes these lipids reactive toward acids and oxidizing agents (1, 2), alters the enzymes required for degradation of the ether bond (14), and, perhaps most importantly, alters the geometry of the lipid and, hence, its biophysical impact on membrane properties (15). PEDS has not been studied in recent years, has not been purified yet, and its coding sequence is still unknown. In this work, we have established a novel assay for quantitative determination of PEDS activities to provide a tool for unraveling the gene for this important enzyme in the future. The assay presented here does not require radiolabeled substrates or lipid extraction procedures after the enzymatic incubation. For the first time, activities in various mouse tissues and mammalian cell lines are compared. Among tissues, we consistently observed the highest activities in kidneys. Previous work had used brain preparations from young rats as source for PEDS activity (16). In our hands, however, the activity in both cerebrum and cerebellum from adult mice was below the detection limit. This may have been caused by the fact that we used tissues from adult animals, because the activity has been shown to be high in brains from newborn rats and then decline rapidly with age (13). We could indeed detect the activity in brains from 7-day-old mice. Spiking experiments ruled out that the low activity in many of the tested mouse tissues was the result of the competition of the labeled substrate by high levels of endogenous unlabeled substrate in the tissues. Using up to 8-fold more lyso-substrate [I] also did not result in measurable PEDS activities in adult mouse cerebellum or in adult mouse liver (data not shown).

We found robust PEDS activity in all 21 cell lines examined, which, except for RAW-12, were at least one order of magnitude higher as compared with the highest activities found in mouse tissues. The plasmalogen content of the cells, however, was in the same order of magnitude as for the mouse tissues. A possible explanation for this might be that the strongly proliferating tumor cell lines need a higher plasmalogen biosynthesis rate as compared with the tissues to reach a certain steady state level.

The results for the cell lines RAW-12 and RAW-108 and the RAW264.7 parent line agree well with a previous report (8). RAW-12 and RAW-108 had been selected for a deficiency in ether lipid biosynthesis (8) and have a low plasmalogen content due to a deficiency in glycerone phosphate *O*-acyltransferase (GNPAT; EC 2.3.1.42), the first enzyme in peroxisomal ether lipid biosynthesis. Interestingly, the additional PEDS deficiency of RAW-12 cells does not result in altered steady state plasmalogen levels in RAW-12 versus RAW-108, and only becomes manifest after feeding of alkylglycerols.

We checked the expression of the peroxisomal ether lipid biosynthetic enzymes (17) in the cells lines in GEO data sets of the NCBI. The GSE accession numbers refer to the data set cited. We found that the exceptionally low plasmalogen levels that we detected in MCF7 go along with a selectively impaired expression of alkylglycerone phosphate synthase (AGPS; EC 2.5.1.26, GSE94479), which is in line with results from previous biochemical investigations (18). HEP G2 cells, for which we also found rather low plasmalogen content, are selectively impaired in fatty acyl-CoA reductase 1 (FAR1; EC 1.2.1.84, GSE83551). Thus, at least in these cells, the low plasmalogen levels appear to be determined primarily by impairment of critical factors other than PEDS activity.

Another novel finding of our work is that PMSF and thiols are inhibitors of PEDS. The facts that the potent disulfide bond reducing agent, TCEP, did not inhibit the reaction and that DTT tended to be a stronger inhibitor than DTE or mercaptoethanol suggest a specific interaction of these inhibitors with the enzyme. Thiols have also been found to inhibit sphingolipid C4 desaturase (EC 1.14.19.17) (19) and decarbonylation of aldehydes to alkanes (20), which today is known to be carried out by deformylating aldehyde oxygenase (EC 4.1.99.5). Both reactions are mediated by di-iron cluster-containing enzymes. We speculate, therefore, that PEDS might share the mechanism with these two reactions, i.e., might also be a di-iron center containing protein.

A puzzling finding already reported in the literature is that, although it is assumed that the 1-*O*-alkyl 2-acyl *sn*-glycerophosphoethanolamines are the substrates for PEDS, the assay works better with the 2-lyso-compound (5, 6). This lyso-compound is not part of the agreed biosynthetic scheme for the biosynthesis of plasmalogens (1). It has been suggested that the acylation process itself may position the lyso-substrate [I] into the membrane to be acylated there to the substrate [II] and, in this way, being better accessible for the desaturase (21). Our findings with the inhibition of the desaturase reaction by inhibiting the acylation of the lyso-substrate [I] with PMSF and SKF 98625 are compatible with this view. In contrast to a report in the literature (16), we have not seen an influence of CoA, fatty acids, or ATP on the acylation reaction. These properties, together with the inhibition of the enzyme by PMSF and SKF 98625 indicate that CoA-independent transacylase (EC 2.3.1.147), an enzyme with still unknown coding sequence, may perform the acylation reaction observed in our microsomal preparations. Although the assay with the lyso-substrate [I] results in a coupled reaction of transacylase followed by the desaturase, we favored the lyso-substrate [I] over the substrate [II] due to the 7-fold higher assay sensitivity obtained.

The neutral lipophilic strongly fluorescent pyrene residue has been used to investigate the metabolism of fatty acids (22), ether lipid metabolism in intact cells (23), the cleavage of alkylglycerols (24), and the oxidation of fatty aldehydes (10, 25). Our work presented here shows another application of this powerful tool for the study of lipid metabolism. We hope that the novel method we developed will help us in our search for the enigmatic gene encoding PEDS, using approaches that have proved successful in our quest for the alkylglycerol monooxygenase gene (26).
